# Magnetic Tracking and Electrocardiography-Guided Tip Confirmation System Versus Fluoroscopy for Placement of Peripherally Inserted Central Catheters: A Randomized, Noninferiority Comparison

**DOI:** 10.1007/s00270-020-02551-0

**Published:** 2020-06-17

**Authors:** V. Mack, D. Nißler, D. Kasikci, A. Malouhi, R. Aschenbach, U. Teichgräber

**Affiliations:** 1 Department of Radiology, Jena University Hospital, Am Klinikum 1, 07747 Jena, Germany; 2 Institut für Diagnostische Und Interventionelle Radiologie, Universitätsklinikum Jena, Am Klinikum 1, 07747 Jena, Germany

**Keywords:** Central venous catheterization, Electrocardiography, Fluoroscopy, Peripheral catheterization, Radiation exposure

## Abstract

**Purpose:**

To determine whether the use of a magnetic tracking and electrocardiography-guided catheter tip confirmation system (TCS) is safe and noninferior to fluoroscopy concerning positioning accuracy of a peripheral inserted central catheter (PICC).

**Methods:**

In this prospective, randomized, single-center study, adult patients scheduled for PICC insertion were assigned 1:1 either to TCS or fluoroscopy. The primary objective was a noninferiority comparison of correct PICC tip position confirmed by X-ray obtained immediately after catheter insertion. Time needed for PICC insertion and insertion-related complications up to 14 days after the procedure were secondary outcomes to be assessed for superiority.

**Results:**

A total of 210 patients (62.3 ± 14.4 years, 63.8% male) were included at a single German center between June 2016 and October 2017. Correct PICC tip position was achieved in 84 of 103 TCS (82.4%) and 103 of 104 fluoroscopy patients (99.0%). One-sided 95% lower confidence limit on the difference between proportions was −23.1%. Thus, noninferiority of TCS was not established (*p* > 0.99). Insertion of PICC took longer with TCS compared to fluoroscopy (8.4 ± 3.7 min vs. 5.0 ± 2.7 min, *p* < 0.001). Incidence of complications within a mean follow-up of 5.0 ± 2.3 days did not differ significantly between groups.

**Conclusion:**

Noninferiority of TCS to fluoroscopy in the incidence of correct PICC tip position was not reached. Ancillary benefit of TCS over fluoroscopy including less radiation exposure and lower resource requirements may nonetheless justify the use of TCS. The study is registered with Clinical.Trials.gov (Identifier: NCT02929368).

## Introduction

Peripherally inserted central catheters (PICC) are widely used to provide central venous access for infusion of irritant drugs, parenteral nutrition, long-term drug administration, repeat blood sampling, monitoring of circulatory functions, or as alternative for patients with poor peripheral access.

PICC insertion, particularly in the case of malposition, however, may cause complications including malfunction, cardiac arrhythmia, or tamponade [1]. In addition, tip migration due to malposition increases the risk of venous thrombosis and infection [2]. Reposition or reinsertion of PICC in the case of malposition is accompanied with risk, delay of patient treatment, and costs.

Currently, there are several approaches of PICC insertion. The classic technique of blind PICC insertion toward a target tip position according to an earlier determined distance between puncture site and an anatomical landmark can be conducted at bedside. However, malposition of PICC is given as 20–76% [3, 4] and X-ray confirmation is needed prior to use of the catheter. In contrast, PICC placement under fluoroscopic control goes along with nearly 100% proper tip position [5, 6]. However, radiation exposure and staff requirements are increased. From this, it might be expected that the use of a magnetic tip confirmation system (TCS) including real-time electrocardiography (ECG) guidance that can be used at bedside would provide decreased incidence of PICC malposition compared to blind insertion and decreased costs and radiation exposure compared to fluoroscopy. Incidence of accurate tip placement with TCS ranges from 79.5 to 100% [7–9]. However, to date, no results from direct comparisons between TCS and fluoroscopy are available.

Our study aimed to compare incidences of accurate PICC tip position between TCS and fluoroscopy and to examine whether TCS is noninferior to fluoroscopy. In addition, we sought to investigate early PICC insertion-related complications within 14 days after PICC implantation.

## Methods

### Study Design and Setting

The study was a prospective, randomized, open-label, single-center, investigator initiated, noninferiority trial that sought to determine whether PICC insertion using TCS is noninferior to fluoroscopy regarding the incidence of correct PICC tip position confirmed by chest X-ray. Eligible patients were allocated 1:1 to either TCS or fluoroscopy by means of computer-generated randomization. The study is registered with Clinical.Trials.gov (Identifier: NCT02929368).

### Study Population and Technique of PICC Insertion

Patients were eligible for inclusion if they were at least 18 years of age and had a medical indication for PICC insertion. Exclusion criteria were systemic infection, infection including puncture site, and known allergy to materials used. Additionally, cardiac arrhythmia including atrial fibrillation, severe tachycardia, or paced rhythm was exclusion criterion because it could interfere with interpretation of the *P*-wave morphology when using the TCS system. Patients were recruited from different medical departments of the center.

PICC insertion was conducted either in the radiology department (fluoroscopy group) or at bedside (TCS group). However, both approaches were carried out by experienced interventional radiologists. Initially, ultrasound was used to identify a suitable vein in the upper arm, preferably the right basilic vein. The vein was punctured, and a guidewire inserted. In the control group, the guidewire was advanced under fluoroscopic control through the axillary and subclavian vein toward the target position near the cavoatrial junction. Length of the guidewire was measured to determine the required PICC length. Subsequently, an introducer sheath was inserted over the wire, the guidewire was removed, and the PICC catheter inserted through the introducer sheath and then advanced under fluoroscopic control to the predetermined length. The same PICC kit type in different sizes was used in all patients.

**Fig. 1:**
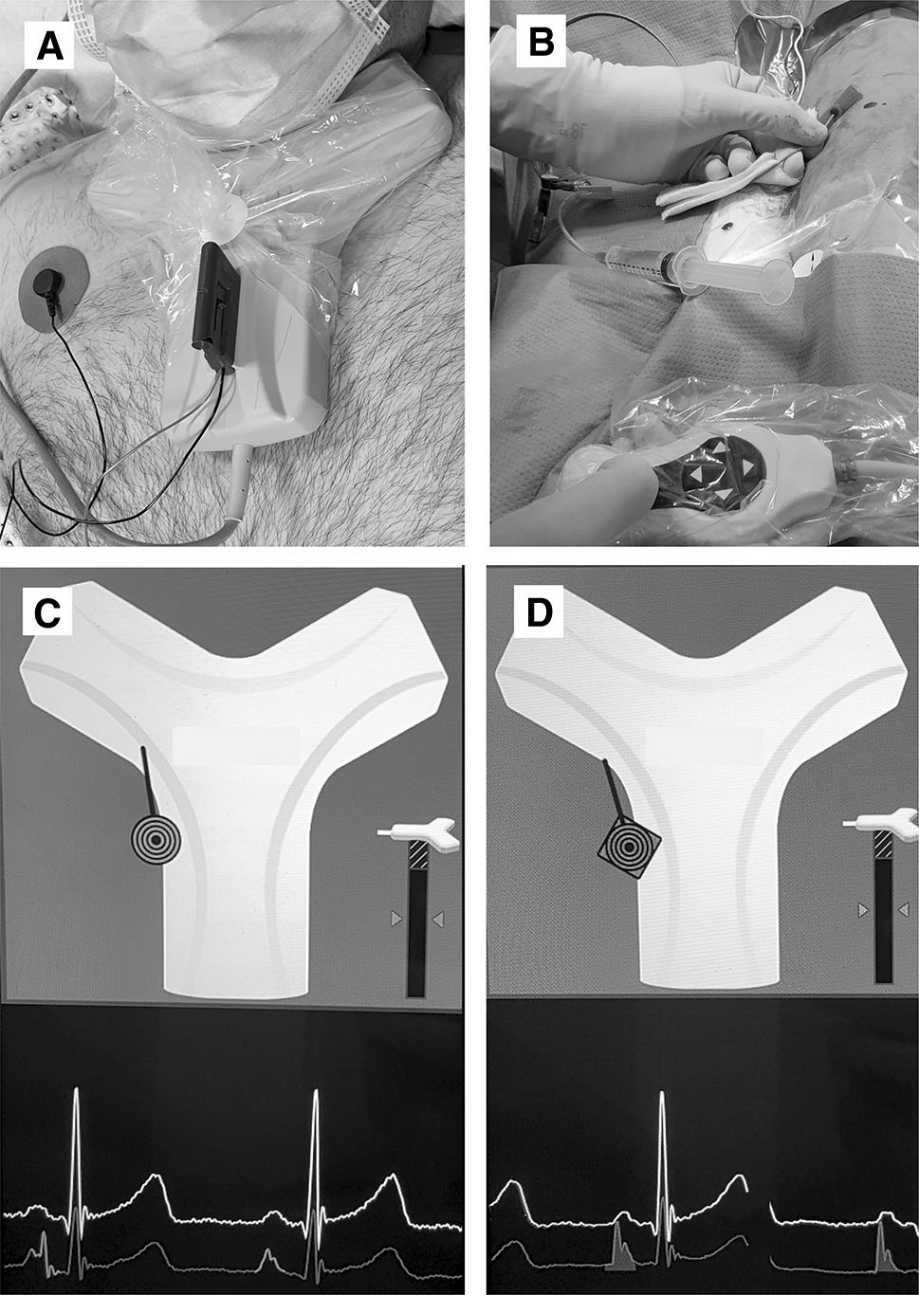
The Y-shaped electromagnetic sensor is positioned on the patient’s sternum. Two leads pick up external ECG waveforms **(A)**. The PICC catheter is then inserted through the sheath into the brachial vein and advanced toward the cavoatrial junction **(B)**. Magnets in the stylet of the catheter tip generate a field that is detected by the sensor and thus can be tracked in real time on the display (*circle*). The display also shows ECG waves from skin and catheter tip **(C)**. *Circle* turns into rhombus and *p*-waves are highlighted when the catheter tip reaches the cavoatrial junction **(D)**

In the investigational group, the Sherlock 3CG^®^ TCS (including single-use PowerPICC SOLO catheter with Sherlock 3CG^®^ tip positioning system stylet, Becton, Dickinson and Company, Franklin Lakes, NJ, USA) was used for magnetic tracking of the PICC tip and real-time ECG confirmation of the tip position during insertion (Fig. 1). The Sherlock sensor was placed on the patient’s sternum to detect the magnetic field of a magnet, encapsulated within the PICC stylet, indicating path of the catheter and enabling passive navigation. Simultaneously, ECG *P*-wave changes with the approach of the tip toward the right atrium and thus, indicating PICC tip location in relation to the sinoatrial node. The maximum ECG *P*-wave anatomically corresponds with the cavoatrial junction. The PICC was inserted with the guidewire inside to support proper placement. After PICC insertion, the guidewire was removed. Signal intensity was documented in all patients in the TCS group.

Finally, in both groups, the PICC was attached on the arm with a seamless stabilization device (StatLock™, Bard Access Systems, Becton, Dickinson and Company, Franklin Lakes, NJ, USA). Placement of the catheter was confirmed by obtaining chest X-ray.

### Study Outcome Measurements

Correct PICC tip position was defined as within the mid to lower superior vena cava, at the level of the cavoatrial junction, or within the upper portion of the right atrium, corresponding to 1.5 vertebral body units (approximal 3 cm) from the tracheal carina on chest X-ray obtained immediately after insertion. Time for PICC insertion started from introduction of the guidewire into the vein and ended with stabilization of the catheter on the arm. In order to assess catheter-related complications, patients were followed up within 2 weeks after PICC insertion. Patients were considered lost to follow up at withdrawal of the catheter.

### Statistical Analysis

Sample size was calculated based on results from a post-market clinical trial on Sherlock 3CG TCS initiated by the manufacturer and described in manufacturers instruction for use that reported acceptable PICC placement in 99.1% of patients. We assumed an incidence of 98.0% success using fluoroscopy. Given a noninferiority margin of 5% and a one-sided 5% level of significance, a sample size of 210 patients was sufficient to establish noninferiority with 98% power. At a significance level of 0.05, noninferiority of TCS compared to fluoroscopy could be claimed if the lower bound of the one-sided 95% CI for the difference in proportions of proper tip position was greater than −5%. The calculation allowed for 10% dropout. Analysis was performed based on intention to treat. The test for noninferiority was only applied for the primary endpoint. All other analyses were tests of superiority. Logistic regression was applied to determine odds of malposition in obese patients. To assess consistency in the effect of TCS across selected patient characteristics, a post hoc subgroup analysis was performed. Continuous variables are reported as means and standard deviations, and categorical variables are presented as counts and percentages. Variables were compared by means of Mann–Whitney *U* test or Fisher’s exact test. A two-sided value of *p* < 0.05 was considered to indicate statistical significance. Analysis was performed using XLSTAT (Version 2015.6.01.24026, Addinsoft, Paris, France).

## Results

### Study Population and Procedure

**Fig. 2:**
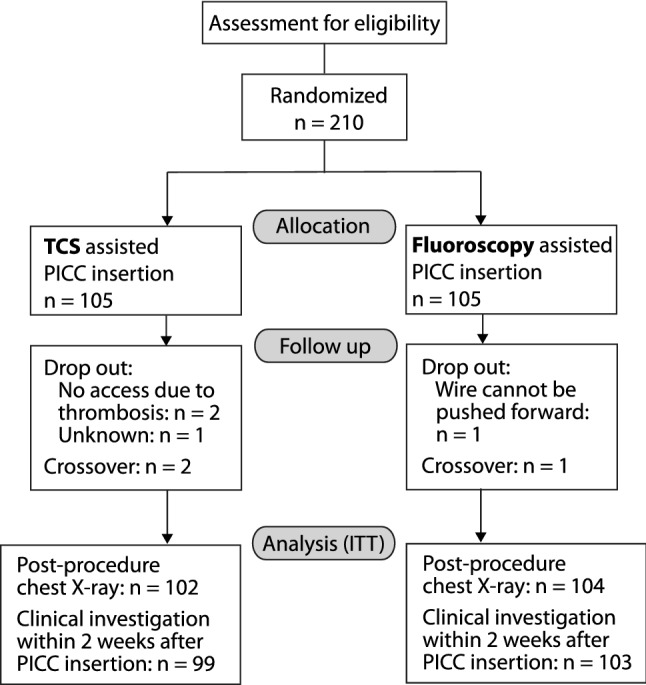
Patient flow. *ITT* intention to treat, *PICC* peripherally inserted central catheter, *TCS* tip confirmation system

From June 2016 to October 2017, 210 patients were enrolled at a single German center and randomly assigned to undergo PICC implantation under either fluoroscopic control or by means of magnetic tracking and ECG-guided TCS (Fig. 2). Patient characteristics of the two groups were well matched at baseline (mean age 62.3 ± 14.4 years, 134 [63.8%] male, mean BMI 25.7 ± 6.1 kg/m^2^). Insertion of PICC using TCS took significantly longer compared to fluoroscopy (8.4 ± 3.7 min vs. 5.0 ± 2.7 min, *p* < 0.001) (Table 1).

**Table 1:** Patient and procedure characteristics

Characteristics	TCS-assisted PICC insertion, *n* = 105		Fluoroscopy-assisted PICC insertion, *n* = 105		*p* value
Age, years	60.6	± 13.8	64.1	± 13.0	*p* = 0.07
Male	71	(67.6)	63	(60.0)	*p* = 0.25
BMI	26.0	± 6.5	25.4	± 5.7	*p* = 0.37
BMI ≥ 30 kg/m^2^	22/103	(21.4)	17/104	(16.3)	*p* = 0.36
Platelet count, 10^9^/l	266	± 135	248	± 124	*p* = 0.43
Partial thromboplastin time, s	35.7	± 45.1	29.9	± 13.6	*p* = 0.44
Time required for PICC insertion, min	8.4	± 3.7	5.0	± 2.7	*p* < 0.001

Values are mean ± standard deviation or counts (percentage)

*BMI* body mass index, *TCS* tip confirmation system

### Primary Outcome

**Fig. 3:**
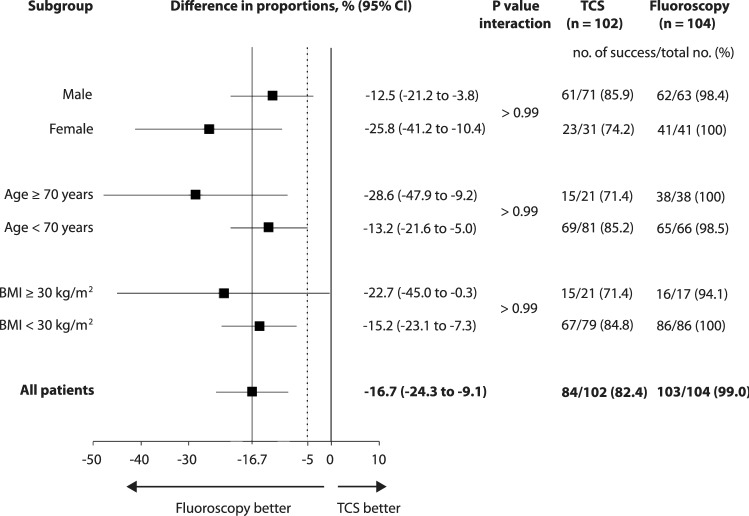
Subgroup analysis of correct tip location (primary endpoint) depending on patient characteristics. *Continuous lines* show overall treatment effect point and no effect point, respectively. *Dotted line* indicates noninferior margin. *BMI* body mass index, *TCS* tip confirmation system

Incidence of correct PICC tip position was 82.4% (84 of 102) in the TCS group and 99.0% (103 of 104) in the fluoroscopy group. The lower bound of a one-sided 95% confidence interval (CI) of −23.1% difference in proportions of proper tip position was below the prespecified boundary of −5%. Thus, noninferiority of TCS compared to fluoroscopy was not established (*p* value for noninferiority: > 0.99). The two-sided 95% CI was entirely below the noninferior margin, and therefore demonstrated inferiority of TCS over fluoroscopy (−16.7% [95% CI −24.3 to −9.1%], *p* < 0.001) (Fig. 3). Logistic regression revealed an increased odds for malposition in obese patients (odds ratio 2.9 [95% CI 1.1 to 7.9], *p* = 0.04). However, age, sex, and BMI did not interact with the effect of TCS (Fig. 3). Five of 18 cases of incorrect tip position in the TCS group (27.8%) were associated with a weak signal of the TCS. In 12 of 17 cases of weak TCS signal, a correct tip position was achieved based on ECG guidance alone. Weak signal was not significantly associated with obesity (odds ratio 1.3 [95% CI 0.4 to 4.6], *p* = 0.67).

### Complications

Mean clinical follow-up time was 5.0 ± 2.3 days. Within this period, incidence of complications including bleeding, pain, allergic reaction, infection, venous thrombosis, nerve damage, and catheter malfunction did not differ between groups (Table 2). All bleeding events were minor access site bleedings that were resolved by compression. Pain was mild to moderate and resolved within a few days after PICC insertion. Allergic reactions were due to an antimicrobial film dressing and symptoms gradually disappeared upon removal of the dressing. Local puncture site infection in two patients per group was successfully treated with cold compresses. In none of the patients, catheter malfunction, thrombosis, or nerve damage occurred.

**Table 2:** Complications within 2 weeks after PICC^1^

Complication	TCS-assisted PICC insertion, *n* = 99		Fluoroscopy-assisted PICC insertion, *n* = 103		*p* value
Bleeding event^2^	8	(8.1)	13	(12.6)	*p* = 0.36
Pain^3^	8	(8.1)	12	(11.7)	*p* = 0.48
Allergic reaction^4^	0	(0.0)	3	(2.9)	*p* = 0.25
Infection^5^	2	(2.0)	2	(1.9)	*p* > 0.99
Thrombosis	0	(0.0)	0	(0.0)	
Nerve damage	0	(0.0)	0	(0.0)	
Catheter malfunction	0	(0.0)	0	(0.0)	

Values are counts (percentage)

^1^Mean follow-up: 5.0 ± 2.3 days

^2^All bleedings were minor access site bleedings

^3^Mild to moderate pain

^4^Due to antimicrobial film dressing

^5^Local wound infection, no septicemia

*PICC* peripherally inserted central catheter*, TCS* tip confirmation system

## Discussion

This randomized, noninferiority trial compared incidences of correct PICC tip position in adult patients supported by either TCS or fluoroscopy. Inferiority of TCS compared to fluoroscopy was established. No difference of catheter-related complications up to 14 days after PICC insertion occurred. Although, in our study, PICC implantations were conducted by experienced radiologists in order to ensure comparability, PICC insertion with TCS assistance is suitable to be performed by specialized nurses, and thus may go easy on resources.

Incidence of proper tip position in our study was in line with retrospectively acquired findings from Johnston et al. (79.5%) [8] and Yamagashi et al. (83.8%) [9]. However, even intracavitary ECG guidance alone was shown to provide 89.2% incidence of accurate tip position [10]. All trials on TCS excluded patients not in sinus rhythm but only the latter two applied the stricter definition of proper tip position within the lower superior vena cava or at the level of the cavoatrial junction. Based on the results of these studies, we deduced to treat previous information on Sherlock 3CG TCS from not peer-reviewed sources that report considerably higher success rates of up to 100% with caution.

### Predictors of Malposition

Cardiac arrhythmia including atrial fibrillation, severe tachycardia, or paced rhythm was exclusion criterion in our study because *P*-wave could not be used as reliable parameter for ECG guidance. However, according to manufacturer, an altered or pacemaker-driven cardiac rhythm is considered limiting but not contraindicated. Proper PICC tip location should, however, be confirmed with chest X-ray. Regarding a 10% prevalence of atrial fibrillation in patients from 65 years of age [11], we consider the limitation as relevant. Nonetheless, Gao et al. found no difference in accuracy between ECG or X-ray tip position verification in patients with atrial fibrillation [12]. Moreover, a recent pilot study provided promising results from modified intracavitary ECG considering *f*-waves in successive TQ-segments for catheter tip location in atrial fibrillation patients [13].

In our study, a weak signal from the magnetic stylet of the PICC tip to the display monitor impeded tip localization and navigation in about 16% of TCS patients resulting in final malposition in one-third of them. Although obesity increased the odds of malposition with both TCS and fluoroscopy, it did not interact with the effect of TCS nor was it associated with a weak signal. In order not to lose the signal, PICC had to be advanced slowly and carefully, resulting in prolonged insertion time compared to fluoroscopy. However, latter entails a radiation exposure of about 60 µGym^2^ [14]. Finally, based on our results, confirmatory chest X-ray should be conducted at least in those TCS patients without an identifiable ECG *P*-wave, in particular in the case of weak TCS signal.

Although, our study did not prove any interaction of sex and age with the effect of TCS, female and elderly patients tended to benefit more from fluoroscopy. Whether osteoporosis-related reduction in the vertebral body height might affect radiographic assessment [9] remains to be clarified.

### Consequences of Malposition

Malposition may increase the risk of catheter tip migration and thus promote thrombosis and infection [2, 6, 15] particularly if the PICC tip is located in the upper two-thirds of the superior vena cava. Venous thrombosis, one of the most serious complications of PICC implantation, may result in bloodstream infection or pulmonary embolism. PICC-related thrombosis occurs at a frequency of 0.5–20% [16, 17]. Pan et al. identified being bedridden for > 72 h, increased levels of D-dimer, and comorbidities as risk factors. The authors reported on 73% cases of thrombosis that occurred within the first, and of 19% cases within the second week after PICC insertion. In our study, no thrombosis was detected up to 14 days after PICC insertion. In patients who experienced infection, no malposition was observed.

### Limitations

The short follow-up period of 14 days does not permit final conclusions on safety. However, we focused on insertion-related complications, not on safety of PICC in general. Thus, a relatively short follow-up period might be considered appropriate. In addition, we did not consider morbidity of patients. There might have been different results in critical ill compared to normally hospitalized patients. Requirement for confirmatory chest X-ray in the TCS group was not determined because in our study, chest X-ray was mandatory to evaluate tip position accuracy. No data were available whether PICC had to be adjusted or reinserted in the case of initial malposition. Finally, to date, there is no consistent definition of an optimal catheter tip position. Therefore, malposition rates could have differed if only the lower vena cava and the cavoatrial junction were considered correct. Finally, we did not consider costs.

## Conclusions

TCS for PICC insertion was associated with less tip position accuracy than fluoroscopy. However, it was associated with reasonable success and a similar complication rate. For this reason, we conclude that TCS would be most useful in patients where fluoroscopy cannot be used, bedside placement is necessary, or resources are limited.
